# Long-term trends in anemia prevalence before and after COVID-19 among non-pregnant adults in South Korea, 2010 to 2023

**DOI:** 10.1097/MD.0000000000046295

**Published:** 2026-05-12

**Authors:** Ye Won Kim, Juyeong Kim, Yesol Yim, Seoyoung Park, Hyunjee Kim, Lee Smith, André Hajek, Jiyoung Hwang, Dong Keon Yon

**Affiliations:** a Center for Digital Health, Medical Science Research Institute, Kyung Hee University Medical Center, Kyung Hee University College of Medicine, Seoul, South Korea; b Department of Regulatory Science, Kyung Hee University, Seoul, South Korea; c Department of Precision Medicine, Kyung Hee University College of Medicine, Seoul, South Korea; d Centre for Health Performance and Wellbeing, Anglia Ruskin University, Cambridge, UK; e Department of Public Health, Faculty of Medicine, Biruni University, Istanbul, Turkey; f Department of Health Economics and Health Services Research, University Medical Center Hamburg-Eppendorf, Hamburg Center for Health Economics, Hamburg, Germany; g Department of Pediatrics, Kyung Hee University College of Medicine, Seoul, South Korea.

**Keywords:** anemia, COVID-19, non-pregnant adults, South Korea, trend

## Abstract

Anemia is a global public health concern, yet its long-term trends in the general adult population, particularly during the COVID-19 pandemic, remain unclear. This study analyzed long-term trends and risk factors for anemia among non-pregnant adults including the pandemic. We analyzed data from the Korea National Health and Nutrition Examination Survey conducted on non-pregnant adults. Anemia was defined as a hemoglobin level of <13 g/dL in males and <12 g/dL in females. The prevalence of anemia was assessed across the pre- (2010–2019), intra- (2020–2022), and post-pandemic (2023) using a weighted linear regression, and risk factors were evaluated using multivariable weighted logistic regression. About 68,696 non-pregnant adults (29,074 males [42.32%]) were included in the analysis from 2010 to 2023. The prevalence of anemia showed an increasing trend from 8.49% (95% confidence interval [CI], 8.20–8.78) in pre-pandemic to 11.23% (10.61–11.86) in intra-pandemic and 11.93% (10.86–13.00) in post-pandemic. A significant rise was observed during the intra-pandemic period, with adjusted odds ratio of 1.37 (95% CI: 1.21–1.56) compared to pre-pandemic years. Anemia prevalence tended to rise with age in males, whereas it reached its lowest point in females aged 50 to 59 years. Key risk factors included female sex, older age, underweight, lower household income, and no dietary supplement use. Anemia prevalence has risen steadily over the past decade and further escalated during the COVID-19 pandemic. Although sex-based patterns followed overall patterns, age-related trends varied by sex. These results emphasize the need for age- and sex-specific public health strategies and targeted interventions for vulnerable groups.

## 1. Introduction

Anemia is a major global public health issue, affecting approximately one-quarter of the world’s population as of 2021, and posing substantial personal, societal, and economic burdens.^[1]^ It is characterized by insufficient hemoglobin levels to support the body’s physiological oxygen needs,^[2]^ which can lead to various symptoms including fatigue, weakness, cognitive impairment, and reduced work productivity.^[1]^ Beyond its physiological consequences, anemia serves as an indicator of poor nutritional status and deteriorating health, compromising physical function, psychosocial well-being, and overall quality of life.^[3]^ Accordingly, identifying and managing factors associated with anemia is essential for improving population health outcomes.

Although anemia is most commonly observed in children and pregnant adults,^[4,5]^ non-pregnant adults are also susceptible due to a wide range of contributing factors, including inadequate dietary intake, chronic disease, socioeconomic disadvantage, and lifestyle behaviors.^[6]^ Despite the substantial disease burden, long-term epidemiological data on anemia in non-pregnant adults remain limited. Given the wide-ranging health consequences of anemia and the growing need to address population health inequalities,^[7]^ it is essential to better understand its determinants in this overlooked population group and to inform targeted prevention and management strategies.

The COVID-19 pandemic has introduced widespread disruptions to health systems, socioeconomic conditions, and daily life behaviors across the globe.^[8]^ Changes in employment, economic stability, dietary patterns, physical activity, and access to healthcare may have influenced the prevalence and risk of anemia, particularly among vulnerable groups.^[9,10]^ For example, during strict lockdown periods, many low-income families experienced job loss and food insecurity, leading to decreased intake of iron-rich foods.^[11]^ Additionally, disruptions in routine health checkups meant that cases of anemia went undiagnosed or untreated.^[12]^ Given these potential associations, it is necessary to examine how anemia trends have evolved across different phases of the pandemic.

Therefore, this study aimed to assess long-term trends in anemia prevalence among non-pregnant adults in South Korea, using nationally representative data from the Korea National Health and Nutrition Examination Survey (KNHANES) from 2010 to 2023. We examined changes in anemia prevalence across pre-, intra-, and post-pandemic periods, and identified key risk factors associated with anemia. The findings from this study are expected to provide valuable evidence to inform targeted public health interventions and future policy planning.

## 2. Methods

### 2.1. Data source

This study used data from the KNHANES, a nationally representative survey conducted by the Korea Disease Control and Prevention Agency from 2010 to 2023.^[13]^ The KNHANES uses a multistage, stratified, probability-based cluster sampling design, with survey areas and households as the primary and secondary sampling units, respectively. The response rates for the 1st (2022) and 2nd (2023) years of the 9th KNHANES cycle were 66.4% and 70.5%, respectively. Sampling weights were applied to ensure national representativeness and to account for differential probabilities of selection and response. A representative sample of 68,696 participants was selected to estimate the prevalence of anemia among non-pregnant adults at the population level. The variables analyzed in this study included sex, age, region of residence, body mass index (BMI), education level, household income, smoking status, household food security status, dietary supplement use, physical activity frequency, and sleep duration. Participants were surveyed across a 14-year period, with the number of individuals as follows: 15,135 in 2010 to 2012; 18,454 in 2013 to 2016; 15,703 in 2017 to 2019; 4482 in 2020; 4554 in 2021; 4794 in 2022; and 5574 in 2023.

The research protocol was approved by the Institutional Review Boards of the Korea Disease Control and Prevention Agency (2010-02CON-21-C, 2011-02CON-06-C, 2012-01EXP-01-2C, 2013-07CON-03-4C, 2013-12EXP-03-5C, 2018-01-03-P-A, 2018-01-03-C-A, 2018-01-03-2C-A, 2018-01-03-5C-A, 2018-01-03-4C-A, and 2022-11-16-R-A). Written informed consent was obtained from all participants prior to their participation. Furthermore, the KNHANES provides public access to its data, which can be utilized as a valuable resource for diverse epidemiological investigations. The study was conducted in accordance with the ethical principles of the Declaration of Helsinki.

### 2.2. Ascertainment of anemia

Hemoglobin concentrations were measured through blood tests to define anemia status. According to the diagnostic criteria established by the World Health Organization (WHO),^[14]^ anemia was defined as a hemoglobin level of <13.0 g/dL in males and <12.0 g/dL in females. To account for the influence of smoking on hemoglobin levels, hemoglobin concentrations were adjusted downward for smokers based on WHO guidelines.^[15]^ The adjustments were applied according to the number of cigarettes smoked per day: 0.3 g/dL for those who did not report their smoking amount or smoked fewer than 10 cigarettes per day, 0.5 g/dL for those smoking 10 to 19 per day, and 0.6 g/dL for ≥ 20 cigarettes per day. These corrections were applied before classification of anemia status.

### 2.3. Covariates

The covariates included in this study were sex (male and female), age group (19–29, 30–39, 40–49, 50–59, 60–69, and ≥ 70 years), region of residence (urban and rural), and BMI group.^[15]^ BMI was classified according to the Asia-Pacific guidelines as underweight (<18.5 kg/m²), normal weight (18.5–22.9 kg/m²), overweight (23.0–24.9 kg/m²), obese (≥25.0 kg/m²), and unknown.^[15]^ Additional covariates included education level (high school or lower and college or higher education), household income (lowest, 2nd, 3rd, and highest quartile), smoking status (current smoker, ex-smoker, nonsmoker, and unknown), household food security status (insufficient and sufficient), dietary supplement use (yes and no), frequency of physical activity (no, 1–3 times per week, ≥4 times per week, and unknown), and average daily sleep duration (<6, 6–9, and ≥ 9 hours). All covariate data were obtained through self-reported questionnaires administered by trained interviewers.

### 2.4. Statistical analysis

The results of this study were presented using qualitative data expressed as proportions or percentages.^[16]^ To ensure accurate population-level estimates, a weighted complex sampling analysis was applied to account for the stratified sampling design and to minimize the influence of differences in participant numbers across survey years. The association between the COVID-19 pandemic and anemia prevalence was evaluated using a weighted linear regression model. We categorized the survey periods into 3 phases. The 1st confirmed case of COVID-19 in South Korea occurred on January 20, 2020, and the WHO declared COVID-19 a global pandemic on March 11, 2020. The South Korean government officially declared the end of the pandemic on May 11, 2023. Accordingly, we defined the pre-pandemic period as 2010 to 2019, the intra-pandemic period as 2020 to 2022, and the post-pandemic period as 2023.^[17]^ β coefficients with 95% confidence intervals (CIs) were calculated and differences in β coefficients were assessed to examine trends in prevalence across the pandemic phases. To identify risk factors associated with anemia, weighted multivariable logistic regression models adjusted for all covariates were employed. The results were presented as adjusted odds ratios (aORs) along with their corresponding 95% CIs. All statistical analyses were performed using SAS software (version 9.4; SAS Institute, Cary) using 2-sided tests, and a *P*-value < .05 was considered statistically significant.

## 3. Results

Of the 108,504 participants surveyed during the study period, adolescents under the age of 19 and pregnant adults were excluded (Figure S1, Supplemental Digital Content, https://links.lww.com/MD/Q810). Among the remaining 86,585 non-pregnant adults, an additional 17,889 participants were excluded due to missing data on household income, food security, dietary supplement use, or hemoglobin levels. As a result, the final survey participants consisted of 68,696 individuals. The weighted characteristics of the study population were as follows: sex (male: 50.14% [95% CI: 49.66–50.62]) and age (19–29 years: 17.40% [95% CI: 16.81–17.98], 30–39 years: 17.21% [95% CI: 16.59–17.83], 40–49 years: 19.76% [95% CI: 19.15–20.36], 50–59 years: 19.60% [95% CI: 19.09–20.11], 60–69 years: 13.65% [95% CI: 13.22–14.08], and ≥ 70 years: 12.39% [95% CI: 11.88–12.90]) (Table 1).

**Table 1:** Weighted characteristics of Korean adults based on data obtained from the KNHANES, 2010 to 2023 (n = 68,696).

Characteristic	Total	Pre-pandemic (2010–2019)	Intra-pandemic (2020–2022)	Post-pandemic (2023)
Overall, n	68,696	49,292	13,830	5574
Sex, weighted % (95% CI)				
Male	50.14 (49.66–50.62)	50.41 (49.95–50.88)	49.91 (49.02–50.80)	50.09 (48.86–51.32)
Female	49.86 (49.38–50.34)	49.59 (49.12–50.05)	50.09 (49.20–50.98)	49.91 (48.68–51.14)
Age, weighted % (95% CI)				
19–29	17.40 (16.81–17.98)	18.38 (17.77–18.99)	17.01 (15.94–18.08)	15.88 (14.22–17.55)
30–39	17.21 (16.59–17.83)	19.14 (18.51–19.77)	16.08 (14.94–17.23)	15.39 (13.68–17.11)
40–49	19.76 (19.15–20.36)	21.26 (20.67–21.85)	18.88 (17.73–20.03)	18.33 (16.72–19.95)
50–59	19.60 (19.09–20.11)	19.57 (19.08–20.06)	19.66 (18.71–20.61)	19.52 (18.17–20.86)
60–69	13.65 (13.22–14.08)	11.52 (11.14–11.89)	14.50 (13.69–15.30)	16.85 (15.49–18.20)
70≤	12.39 (11.88–12.90)	10.14 (9.73–10.54)	13.87 (12.84–14.91)	14.02 (12.47–15.57)
Region of residence, weighted % (95% CI)				
Urban	47.07 (45.67–48.47)	48.57 (47.19–49.96)	46.41 (42.92–49.90)	45.01 (38.98–51.03)
Rural	52.93 (51.53–54.33)	51.43 (50.05–52.81)	53.59 (50.10–57.08)	54.99 (48.97–61.02)
BMI group, weighted % (95% CI)*				
Underweight	4.38 (4.15–4.61)	4.28 (4.05–4.50)	4.39 (3.98–4.81)	4.63 (4.02–5.24)
Normal weight	37.10 (36.57–37.63)	39.49 (38.93–40.04)	35.31 (34.34–36.28)	36.00 (34.57–37.43)
Overweight	22.37 (21.93–22.81)	22.63 (22.18–23.07)	22.27 (21.47–23.07)	21.98 (20.79–23.17)
Obese	35.30 (34.74–35.87)	33.38 (32.83–33.94)	36.79 (35.75–37.83)	36.02 (34.45–37.58)
Unknown	0.85 (0.73–0.97)	0.23 (0.18–0.28)	1.23 (1.00–1.46)	1.38 (0.99–1.77)
Level of education, weighted % (95% CI)				
High school or lower education	45.15 (44.24–46.07)	48.82 (47.91–49.72)	42.34 (40.64–44.03)	43.72 (40.71–46.72)
College or higher education	50.80 (49.84–51.77)	46.77 (45.85–47.70)	53.04 (51.20–54.88)	54.98 (51.92–58.05)
Unknown	4.04 (3.75–4.34)	4.41 (4.11–4.71)	4.62 (4.02–5.22)	1.30 (0.94–1.66)
Household income, weighted % (95% CI)				
Lowest quartile	15.08 (14.46–15.71)	14.94 (14.34–15.54)	15.14 (13.92–16.37)	15.31 (13.37–17.25)
Second quartile	23.38 (22.70–24.06)	25.14 (24.42–25.86)	22.22 (20.99–23.45)	22.11 (20.26–23.96)
Third quartile	29.44 (28.71–30.16)	29.18 (28.48–29.88)	29.52 (28.20–30.84)	29.89 (27.70–32.08)
Highest quartile	32.10 (31.05–33.15)	30.75 (29.80–31.69)	33.12 (31.07–35.16)	32.69 (29.65–35.74)
Smoking status, weighted % (95% CI)				
Smoker	19.61 (19.11–20.11)	22.22 (21.70–22.75)	17.72 (16.80–18.64)	18.21 (16.89–19.53)
Ex-smoker	23.01 (22.57–23.45)	20.96 (20.54–21.38)	24.78 (23.97–25.58)	23.25 (22.06–24.43)
Nonsmoker	56.08 (55.54–56.62)	54.64 (54.11–55.17)	56.95 (55.97–57.94)	57.34 (55.75–58.93)
Unknown	1.30 (1.19–1.41)	2.17 (1.99–2.35)	0.55 (0.40–0.70)	1.21 (0.86–1.55)
Household food security status, weighted % (95% CI)				
Insufficient	2.31 (2.12–2.51)	3.24 (2.95–3.53)	1.89 (1.56–2.22)	1.07 (0.70–1.44)
Sufficient	97.69 (97.49–97.88)	96.76 (96.47–97.05)	98.11 (97.78–98.44)	98.93 (98.56–99.30)
Dietary supplement use, weighted % (95% CI)				
Yes	59.44 (58.78–60.09)	47.07 (46.37–47.77)	67.88 (66.78–68.97)	67.53 (65.83–69.23)
No	40.56 (39.91–41.22)	52.93 (52.23–53.63)	32.12 (31.03–33.22)	32.47 (30.77–34.17)
Physical activity frequency, weighted % (95% CI)				
No	67.41 (66.85–67.97)	69.27 (68.71–69.84)	66.77 (65.77–67.77)	64.30 (62.60–66.00)
1–3 times/wk	16.36 (15.93–16.79)	16.78 (16.32–17.24)	15.72 (14.96–16.48)	17.14 (15.70–18.59)
≥ 4 times/wk	10.79 (10.43–11.14)	9.55 (9.22–9.89)	11.46 (10.80–12.11)	12.11 (11.16–13.06)
Unknown	5.44 (5.10–5.79)	4.39 (4.09–4.69)	6.05 (5.39–6.71)	6.45 (5.42–7.49)
Average sleep duration per day, weighted % (95% CI)				
< 6 hr	13.61 (13.22–14.00)	14.27 (13.86–14.69)	12.91 (12.21–13.61)	13.94 (12.83–15.04)
6–9 hr	76.05 (75.53–76.56)	76.21 (75.69–76.73)	75.28 (74.29–76.27)	77.89 (76.68–79.10)
≥ 9 hr	10.34 (9.95–10.73)	9.51 (9.16–9.87)	11.81 (11.04–12.57)	8.17 (7.32–9.02)

BMI = body mass index, CI = confidence interval, KNHANES = Korea National Health and Nutrition Examination Survey.

* According to Asian-Pacific guidelines, BMI is divided into 4 groups: underweight (<18.5 kg/m^2^), normal weight (18.5–22.9 kg/m^2^), overweight (23.0–24.9 kg/m^2^), and obese (≥25.0 kg/m^2^).

**Figure 1.**
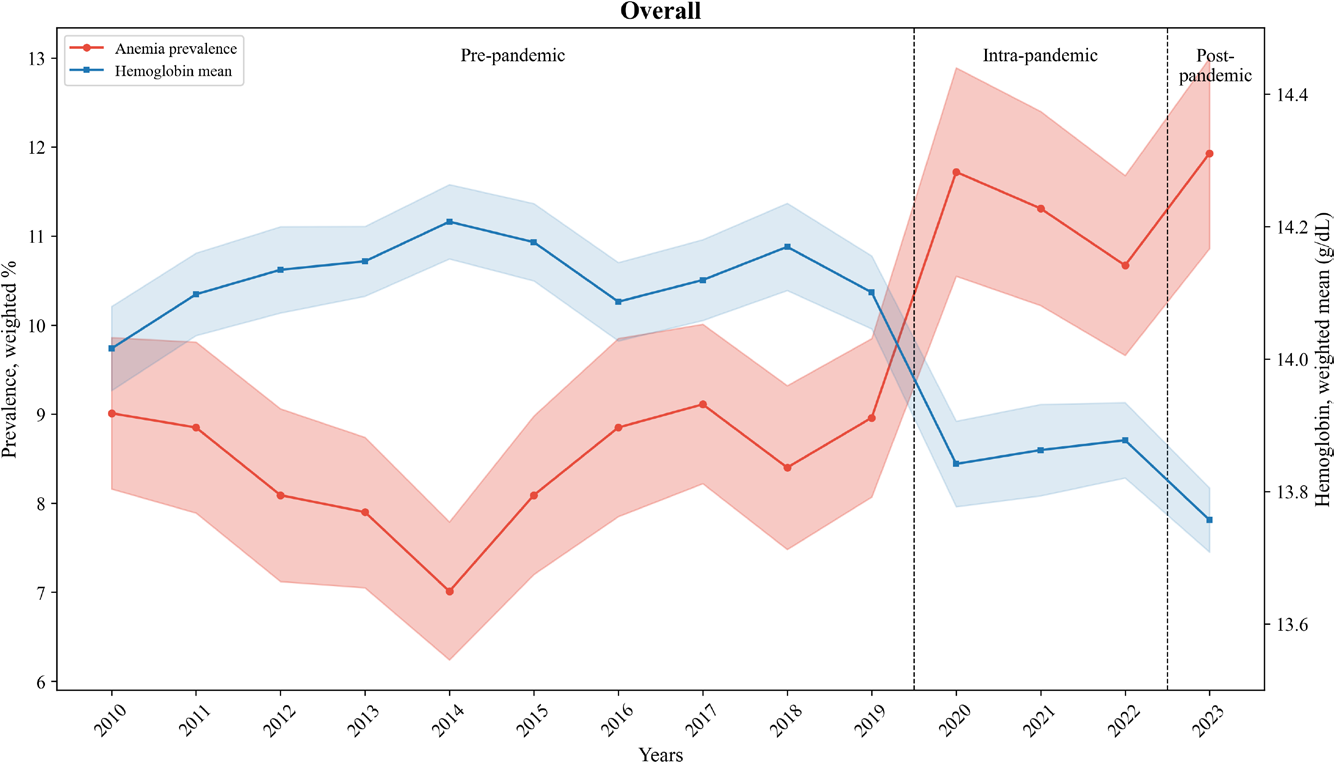
Trends in anemia prevalence and hemoglobin mean from 2010 to 2023 (n = 68,696). Shaded areas represent 95% confidence intervals. Hemoglobin is plotted on the right y-axis.

Table 2 presents the weighted prevalence of anemia and weighted means of hemoglobin across the pre-, intra-, and post-pandemic periods. The underweight group showed a greater increase in prevalence in the post-pandemic period than in the pre-pandemic period: 12.07% (95% CI: 10.50–13.63) in pre-pandemic; 16.50% (13.08–19.92) in intra-pandemic; 24.04% (18.20–29.88) in post-pandemic. Trends in anemia prevalence and hemoglobin mean over the entire study period are shown in Figure 1.

**Table 2:** Weighted trends in the prevalence of anemia pre-, intra-, and post-pandemic (weighted % [95% CI]) based on data obtained from the KNHANES.

Group	Pre-pandemic (2010–2019)	Intra-pandemic (2020–2022)	Post-pandemic (2023)
Anemia			
Overall	8.49 (8.20–8.78)	11.23 (10.61–11.86)	11.93 (10.86–13.00)
Hemoglobin, weighted mean (95% CI)	14.13 (14.11–14.14)	13.86 (13.82–13.90)	13.76 (13.71–13.80)
Sex			
Male	4.33 (4.03–4.64)	6.98 (6.27–7.68)	8.10 (6.96–9.25)
Female	12.73 (12.26–13.20)	15.48 (14.55–16.40)	15.76 (14.05–17.47)
Age			
19–29	5.00 (4.41–5.60)	6.14 (4.86–7.42)	5.49 (3.73–7.25)
30–39	7.47 (6.86–8.08)	8.09 (6.75–9.44)	9.08 (6.92–11.24)
40–49	10.15 (9.46–10.84)	11.29 (9.92–12.66)	13.04 (10.80–15.28)
50–59	5.45 (4.92–5.97)	7.13 (5.97–8.30)	8.22 (6.34–10.09)
60–69	8.73 (8.00–9.45)	10.76 (9.45–12.07)	13.13 (11.20–15.07)
70≤	18.91 (17.90–19.92)	27.35 (25.28–29.43)	24.59 (21.63–27.55)
Region of residence			
Urban	8.19 (7.77–8.60)	10.76 (9.95–11.57)	12.16 (10.37–13.95)
Rural	8.79 (8.38–9.19)	11.65 (10.70–12.59)	11.74 (10.46–13.01)
BMI group*			
Underweight	12.07 (10.50–13.63)	16.50 (13.08–19.92)	24.04 (18.20–29.88)
Normal weight	11.10 (10.60–11.61)	13.85 (12.78–14.92)	14.27 (12.48–16.06)
Overweight	6.96 (6.43–7.49)	11.24 (9.91–12.57)	10.00 (8.19–11.80)
Obese	5.95 (5.55–6.35)	7.55 (6.77–8.32)	8.76 (7.56–9.96)
Unknown	15.04 (7.82–22.27)	27.59 (20.28–34.90)	23.54 (13.34–33.73)
Level of education			
High school or lower education	9.79 (9.36–10.22)	13.74 (12.73–14.74)	15.19 (13.59–16.78)
College or higher education	6.96 (6.57–7.35)	8.47 (7.67–9.28)	8.79 (7.59–9.99)
Unknown	10.41 (8.97–11.84)	19.99 (16.68–23.30)	34.81 (24.98–44.65)
Household income			
Lowest quartile	13.03 (12.25–13.81)	20.55 (18.66–22.45)	20.41 (17.10–23.72)
Second quartile	8.83 (8.26–9.40)	12.15 (10.87–13.43)	13.83 (11.80–15.87)
Third quartile	7.70 (7.21–8.20)	9.28 (8.30–10.26)	9.83 (8.11–11.55)
Highest quartile	6.77 (6.29–7.24)	8.10 (7.12–9.09)	8.58 (7.22–9.94)
Smoking status			
Smoker	2.59 (2.25–2.94)	4.13 (3.30–4.95)	5.62 (3.87–7.37)
Ex-smoker	9.56 (8.90–10.23)	13.27 (12.03–14.52)	15.79 (13.53–18.06)
Nonsmoker	10.42 (10.00–10.84)	12.41 (11.59–13.23)	11.85 (10.44–13.26)
Unknown	10.15 (8.15–12.15)	26.75 (16.52–36.98)	36.25 (25.77–46.74)
Household food security status			
Insufficient	12.97 (11.11–14.84)	18.46 (13.22–23.70)	17.93 (6.56–29.31)
Sufficient	8.34 (8.05–8.64)	11.10 (10.47–11.72)	11.86 (10.80–12.92)
Dietary supplement use			
Yes	8.34 (7.94–8.73)	10.70 (9.98–11.42)	11.79 (10.58–13.00)
No	8.64 (8.23–9.04)	12.37 (11.28–13.46)	12.21 (10.42–14.00)
Physical activity frequency			
No	9.26 (8.91–9.61)	12.07 (11.32–12.81)	12.48 (11.17–13.79)
1–3 times/wk	5.83 (5.27–6.39)	6.96 (5.82–8.11)	8.73 (6.71–10.75)
≥ 4 times/wk	6.79 (5.95–7.63)	9.38 (7.64–11.13)	11.34 (8.95–13.73)
Unknown	10.26 (8.83–11.69)	16.64 (13.90–19.38)	16.01 (12.19–19.83)
Average sleep duration per day			
< 6 hr	9.92 (9.13–10.71)	14.80 (13.00–16.59)	14.05 (11.52–16.58)
6–9 hr	8.04 (7.72–8.37)	10.41 (9.74–11.09)	11.51 (10.38–12.63)
≥ 9 hr	9.98 (9.07–10.89)	12.57 (10.72–14.41)	12.31 (9.12–15.50)

Abbreviations: BMI = body mass index, CI = confidence interval, KNHANES = Korea National Health and Nutrition Examination Survey.

* According to Asian-Pacific guidelines, BMI is divided into 4 groups: underweight (<18.5 kg/m^2^), normal weight (18.5–22.9 kg/m^2^), overweight (23.0–24.9 kg/m^2^), and obese (≥25.0 kg/m^2^).

Table S1 and Figure S2, Supplemental Digital Content, https://links.lww.com/MD/Q810 present the weighted prevalence of anemia from 2010 to 2023. During the pre-pandemic period, the prevalence decreased from 8.65% (95% CI: 8.11–9.19) in 2010 to 2012 to 7.99% (95% CI: 7.54–8.43) in 2013 to 2016, before increasing to 8.82% (95% CI: 8.30 to 9.34) in 2017 to 2019. Subsequently, the prevalence rose to 11.72% (95% CI: 10.55–12.89) in 2020 and 11.93% (95% CI: 10.86–13.00) in 2023. Notably, anemia prevalence significantly increased during the onset of the COVID-19 pandemic. The β-coefficients and β-differences in anemia trends across the pre-, intra-, and post-pandemic periods are presented in Table S2, Supplemental Digital Content, https://links.lww.com/MD/Q810.

Table 3 presents the aORs for risk factors associated with anemia prevalence. Regardless of the COVID-19 period, females consistently exhibited significantly higher anemia prevalence than males: aOR, 3.29 (95% CI: 3.01–3.59) in overall; 3.45 (3.08–3.85) in pre-pandemic; 3.12 (2.75–3.53) in intra-pandemic; and 3.24 (2.66–3.95) in post-pandemic. In individuals aged 70 years and older, aOR for anemia increased from 4.14 (95% CI: 3.50–4.90) in the pre-pandemic period to 4.78 (3.88–5.88) during the intra-pandemic period. The additional identified risk factors for anemia included underweight, lower household income, insufficient food security, and no dietary supplement use. Table S3, Supplemental Digital Content, https://links.lww.com/MD/Q810 presents the aORs of anemia by period. Compared to the 2017 to 2019 period, the prevalence of anemia significantly increased in 2020 (aOR: 1.37 [95% CI: 1.21–1.56]).

**Table 3:** Adjusted odds ratios (weighted % [95% CI]) for vulnerable groups with anemia based on data obtained from the KNHANES.

Variables		Overall (2010–2023)	Pre-pandemic (2010–2019)	Intra-pandemic (2020–2022)	Post-pandemic (2023)
		aOR (95% CI)	aOR (95% CI)	aOR (95% CI)	aOR (95% CI)
Sex	Male	1.00 (ref)	1.00 (ref)	1.00 (ref)	1.00 (ref)
	Female	**3.29 (3.01–3.59**)	**3.45 (3.08–3.85**)	**3.12 (2.75–3.53**)	**3.24 (2.66–3.95**)
Age	19–29	1.00 (ref)	1.00 (ref)	1.00 (ref)	1.00 (ref)
	30–39	**1.62 (1.42–1.85**)	**1.69 (1.45–1.97**)	**1.52 (1.23–1.88**)	**1.54 (1.12–2.12**)
	40–49	**2.41 (2.12–2.73**)	**2.51 (2.15–2.92**)	**2.42 (1.99–2.94**)	**2.30 (1.71–3.09**)
	50–59	**1.32 (1.15–1.52**)	**1.29 (1.09–1.53**)	**1.28 (1.03–1.59**)	1.31 (0.95–1.82)
	60–69	**2.01 (1.75–2.31**)	**1.96 (1.65–2.32**)	**1.93 (1.56–2.37**)	**1.94 (1.42–2.63**)
	70≤	**4.48 (3.91–5.13**)	**4.14 (3.50–4.90**)	**4.78 (3.88–5.88**)	**4.04 (2.95–5.55**)
Region of residence	Urban	1.00 (ref)	1.00 (ref)	1.00 (ref)	1.00 (ref)
	Rural	1.04 (0.97–1.10)	1.04 (0.97–1.13)	1.06 (0.97–1.16)	1.01 (0.86–1.17)
BMI group*	Normal weight	1.00 (ref)	1.00 (ref)	1.00 (ref)	1.00 (ref)
	Underweight	**1.28 (1.12–1.46**)	1.13 (0.96–1.33)	**1.24 (1.01–1.52**)	**1.83 (1.37–2.43**)
	Overweight	**0.66 (0.61–0.72**)	**0.61 (0.55–0.67**)	**0.71 (0.63–0.81**)	**0.70 (0.58–0.83**)
	Obese	**0.53 (0.50–0.57**)	**0.52 (0.48–0.57**)	**0.53 (0.48–0.59**)	**0.54 (0.46–0.63**)
	Unknown	**1.36 (1.02–1.82**)	1.20 (0.65–2.21)	1.25 (0.87–1.79)	1.21 (0.77–1.89)
Level of education	College or higher education	1.00 (ref)	1.00 (ref)	1.00 (ref)	1.00 (ref)
	High school or lower education	0.99 (0.92–1.07)	0.99 (0.90–1.09)	0.96 (0.85–1.08)	1.14 (0.93–1.38)
	Unknown	1.29 (0.94–1.77)	1.39 (0.62–3.11)	1.38 (0.83–2.27)	**1.92 (1.04–3.56**)
Household income	Highest quartile	1.00 (ref)	1.00 (ref)	1.00 (ref)	1.00 (ref)
	Lowest quartile	**1.35 (1.22–1.49**)	**1.28 (1.13–1.45**)	**1.46 (1.25–1.69**)	**1.43 (1.13–1.81**)
	Second quartile	**1.21 (1.11–1.33**)	**1.22 (1.10–1.36**)	**1.19 (1.04–1.37**)	**1.22 (1.01–1.48**)
	Third quartile	**1.11 (1.02–1.21**)	**1.14 (1.03–1.26**)	1.12 (0.98–1.28)	1.04 (0.86–1.26)
Smoking status	Smoker	1.00 (ref)	1.00 (ref)	1.00 (ref)	1.00 (ref)
	Ex-smoker	**3.65 (3.23–4.12**)	**3.81 (3.28–4.44**)	**3.21 (2.69–3.83**)	**3.04 (2.34–3.95**)
	Nonsmoker	**1.65 (1.45–1.88**)	**1.86 (1.59–2.18**)	**1.45 (1.20–1.74**)	1.05 (0.78–1.42)
	Unknown	**1.65 (1.28–2.13**)	**1.69 (1.23–2.33**)	**2.09 (1.34–3.27**)	**2.33 (1.12–4.82**)
Household food security status	Sufficient	1.00 (ref)	1.00 (ref)	1.00 (ref)	1.00 (ref)
	Insufficient	**1.22 (1.04–1.43**)	**1.33 (1.11–1.61**)	1.11 (0.86–1.42)	1.07 (0.56–2.03)
Dietary supplement use	Yes	1.00 (ref)	1.00 (ref)	1.00 (ref)	1.00 (ref)
	No	**1.18 (1.11–1.25**)	**1.23 (1.14–1.33**)	**1.22 (1.12–1.34**)	**1.28 (1.10–1.49**)
Physical activity frequency	No	1.00 (ref)	1.00 (ref)	1.00 (ref)	1.00 (ref)
	1–3 times/wk	**0.84 (0.77–0.92**)	**0.86 (0.77–0.97**)	**0.83 (0.72–0.96**)	**0.77 (0.62–0.97**)
	≥ 4 times/wk	0.99 (0.89–1.11)	1.01 (0.87–1.16)	0.89 (0.75–1.05)	1.12 (0.89–1.41)
	Unknown	1.01 (0.76–1.34)	0.91 (0.40–2.07)	0.81 (0.51–1.29)	0.93 (0.69–1.24)
Average sleep duration per day	6–9 hr	1.00 (ref)	1.00 (ref)	1.00 (ref)	1.00 (ref)
	<6 hr	0.95 (0.88–1.04)	0.97 (0.88–1.08)	1.06 (0.94–1.21)	0.85 (0.70–1.03)
	≥9 hr	1.02 (0.93–1.13)	1.05 (0.94–1.18)	1.02 (0.89–1.17)	1.17 (0.90–1.51)

The figures in bold indicate statistical significance (*P*-value < .05).

The overall multivariable logistic regression model adjusted for sex (male and female), age (19–29, 30–39, 40–49, 50–59, 60–69, and 70≤ years), region of residence (urban and rural), BMI group (underweight, normal weight, overweight, obese, and unknown), level of education(high school or lower education, college or higher education, and unknown), household income(lowest quartile, 2nd quartile, 3rd quartile, and highest quartile), smoking status (current smoker, ex-smoker, nonsmoker, and unknown), household food security status (insufficient and sufficient), dietary supplement use (yes and no), physical activity frequency (no, 1–3 times/week, ≥4 times/week, and unknown) and average sleep duration per day (<6, 6–9, and ≥9 hours).

aOR = adjusted odds ratio, BMI = body mass index, CI = confidence interval, KNHANES = Korea National Health and Nutrition Examination Survey.

* According to Asian-Pacific guidelines, BMI is divided into 4 groups: underweight (<18.5 kg/m^2^), normal weight (18.5–22.9 kg/m^2^), overweight (23.0–24.9 kg/m^2^), and obese (≥25.0 kg/m^2^).

**Figure 2.**
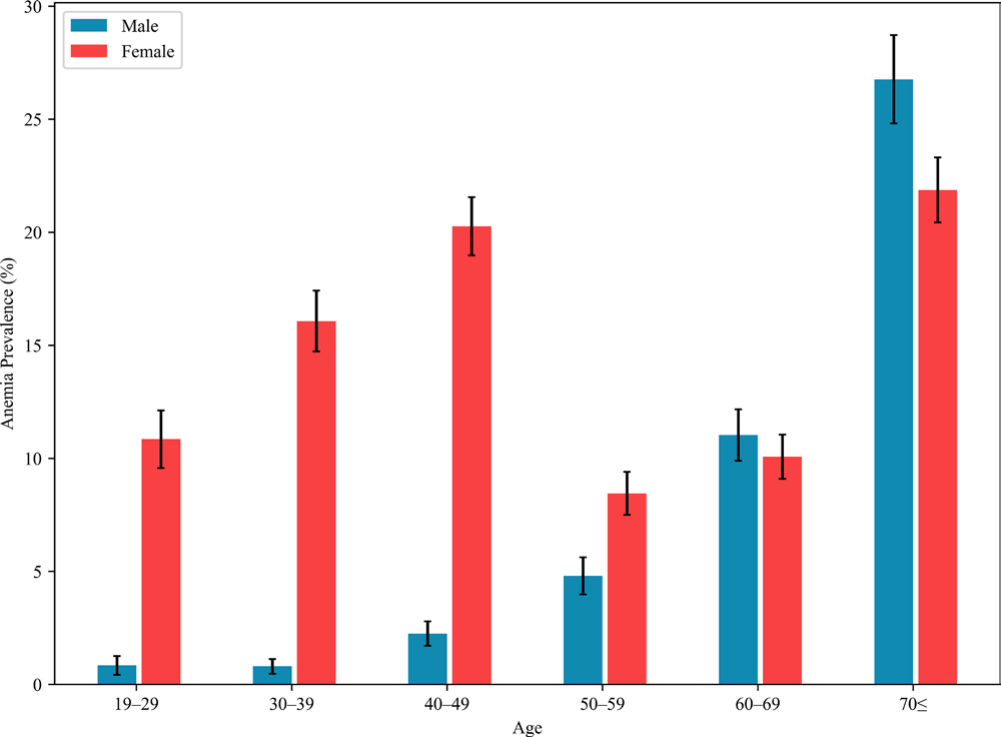
Sex-specific trends in anemia prevalence by age. The error bars indicate the 95% confidence intervals.

Figure 2 and Table S4, Supplemental Digital Content, https://links.lww.com/MD/Q810 display trends in anemia prevalence by sex and age. Among males, prevalence increased with age. Among females, anemia prevalence increased up to the 5th decade of life, declined in the 6th decade of life, and increased again in older age groups. The age group aged 50 to 59 years had the lowest prevalence within the female population. Notably, females in their 5th decade of life had prevalence rates similar to those aged 70 and older, although the latter group exhibited higher rates during the pandemic period.

## 4. Discussion

### 4.1. Key findings

This study comprehensively examined the long-term trend and risk factors associated with the prevalence of anemia among non-pregnant adults from 2010 to 2023, with a particular focus on how the COVID-19 pandemic influenced anemia prevalence. A particularly notable increase in the risk of anemia was observed during the transition from the pre-pandemic years (2017–2019) to the onset of the COVID-19 pandemic in 2020. Overall, anemia prevalence increased with age, but it decreased among females in their 6th decade of life. During the transition from the pre-pandemic to the intra-pandemic period, a significant increase in anemia risk was observed among vulnerable populations, particularly older adults and low-income groups. Additionally, a higher prevalence of anemia was found in groups who did not use dietary supplements.

### 4.2. Plausible underlying mechanisms and comparison to previous studies

In this study, an increasing trend in the prevalence of anemia was observed with the onset of the COVID-19 pandemic, with notable variations according to multiple demographic and lifestyle factors. This trend is presumed to be associated with limited access to healthcare services,^[8]^ nutritional imbalances,^[9]^ and economic inequality,^[18]^ and during the pandemic period, as similarly reported in previous studies. These findings indicate that the structural and societal changes brought about by the pandemic may have adversely affected the management of chronic conditions.^[10]^

Females are considered to be at a significantly higher risk of developing anemia compared to males, primarily due to physiological factors such as menstrual blood loss and increased iron demands during pregnancy and childbirth.^[2]^ Our study is consistent with previous research showing that anemia is more prevalent in females than in males.^[1]^ Additionally, the prevalence of anemia showed a positive association with age,^[6]^ with particularly high rates observed among older adults.^[19]^ This may be attributed to a combination of factors such as decreased iron absorption with aging, the presence of chronic diseases, and reduced nutrients. This is consistent with numerous studies reporting a high prevalence of anemia in older adults.^[19,20]^ Interestingly, among females, the prevalence of anemia temporarily decreased in their 6th decade before rising again in older age groups. This pattern may be explained by the onset of menopause, during which menstruation ceases, and iron loss is reduced, temporarily lowering the risk of anemia. However, as aging progresses, factors such as chronic diseases, poor nutrient absorption, and general health decline likely contribute to a renewed increase in anemia prevalence. This age-related trend aligns with previous studies^[21]^ and highlights the role of physiological changes in anemia development among older adults.^[22]^

In addition, nonuse of dietary supplements was associated with a higher prevalence of anemia. The prevalence of anemia was higher among participants who did not use dietary supplements. Iron, folate, and vitamin B12 are essential for erythropoiesis and inadequate intake or absorption of these nutrients can lead to anemia.^[23]^ Consistent with this biological rationale, our findings align with prior evidence showing that iron supplementation improves hemoglobin concentrations and reduces anemia among affected populations.^[24]^

### 4.3. Clinical and policy implications

In this study, we observed an increasing trend in the prevalence of anemia during and after the COVID-19 pandemic. We identified population groups vulnerable to anemia, including females, older adults, low-income individuals, those with insufficient dietary security, and those did not use dietary supplements.^[25]^ These findings highlight the need for continuous and systematic management of chronic diseases, including anemia, even during public health crises. Anemia results from a complex interplay of factors such as underlying diseases, nutritional status, and lifestyle habits.^[7]^ Therefore, regular health monitoring and personalized nutrition and health counseling are essential.^[26]^ It is important to identify potential high-risk groups at an early stage and implement a prevention-centered approach to mitigate disease progression. Individuals in low-income and nutritionally vulnerable populations may have reduced iron stores and deficiencies in essential nutrients required for hematopoiesis, such as protein, folic acid, and vitamin B12.^[27,28]^ Therefore, from a policy perspective, it is essential to expand food and nutrition assistance for vulnerable populations and strengthen health education. Such integrated and proactive interventions at both the medical and policy levels may help restore health equity in the post-pandemic era and reduce the societal burden of chronic diseases.^[29]^

### 4.4. Strengths and limitations

This study has several limitations. First, anemia was defined solely by hemoglobin levels, which prevented differentiation of specific anemia types and may limit the precision of risk factor analyses. However, hemoglobin is widely recognized and remains a practical indicator in large-scale studies, making it a useful measure for identifying anemia and enabling comparisons across different populations. Second, the adjusted hemoglobin levels of smokers may not be suitable for identifying anemia. A previous study has shown that chronic carbon monoxide exposure leads to higher hemoglobin levels in smokers.^[30]^ Smoking results in an increase in carboxyhemoglobin, the inactive form of hemoglobin, which compensatory raises the hemoglobin concentration. To address this issue, the smoking adjustment factor recommended by the WHO was applied.^[14]^ However, the validity of this adjustment remains controversial. In addition, further efforts are needed to establish blood testing methods specifically tailored to smokers, in order to improve the accuracy of anemia diagnosis in this population. Third, this study used the variable “use of dietary supplements for more than 2 weeks in the past year” to examine its association with anemia prevalence. However, this measure did not indicate whether the products contained iron or whether they were taken to prevent or treat anemia. This limitation reflects the lack of information on supplement composition, dose, duration, and intended use. Moreover, classifying exposure using a short-term threshold provides limited insight into potential benefits. Accordingly, the association between dietary supplements use and anemia should be interpreted with caution. Finally, this cross-sectional study does not allow for causal inference, as it does not account for the temporal order of events. Nevertheless, it identified significant associations between anemia and various factors by analyzing multiple variables at a single point in time. Further longitudinal studies are needed to establish causality.

This study analyzed long-term trends in the prevalence of anemia in the Korean population using 14 years of data from the KNHANES, spanning from 2010 to 2023. This allowed for the evaluation of the impact of the COVID-19 pandemic on anemia prevalence. Weighted analyses were applied to ensure national representativeness based on a large-scale, population-based sample, enabling the production of reliable and generalizable estimates. These analyses also allowed for the identification of vulnerability factors associated with anemia and offer a valuable foundation for the development of future prevention and management strategies.

## 5. Conclusion

This study analyzed long-term trends in anemia prevalence and examined how the COVID-19 pandemic influenced adults (excluding pregnant adults), using nationally representative data from 2010 to 2023. The risk of anemia increased with the onset of the COVID-19 pandemic compared to the pre-pandemic period, and the study identified the following as characteristics of population groups vulnerable to anemia: female sex, older age, underweight status, lower household income and food security, and no dietary supplement use. These findings underscore the importance of proactive and targeted intervention strategies for high-risk groups and offer essential evidence to inform the development of continuous monitoring systems and preventive public health policies.

## Author contributions

**Conceptualization**: Ye Won Kim, Juyeong Kim, Dong Keon Yon.

**Data curation**: Ye Won Kim, Juyeong Kim, Dong Keon Yon.

**Formal analysis**: Ye Won Kim, Juyeong Kim, Dong Keon Yon.

**Funding acquisition**: Ye Won Kim, Juyeong Kim, Dong Keon Yon.

**Investigation**: Ye Won Kim, Juyeong Kim, Dong Keon Yon.

**Methodology**: Ye Won Kim, Juyeong Kim, Dong Keon Yon.

**Project administration**: Ye Won Kim, Juyeong Kim, Dong Keon Yon.

**Resources**: Ye Won Kim, Juyeong Kim, Dong Keon Yon.

**Software**: Ye Won Kim, Juyeong Kim, Dong Keon Yon.

**Supervision**: Dong Keon Yon.

**Validation**: Ye Won Kim, Juyeong Kim, Dong Keon Yon.

**Visualization**: Ye Won Kim, Juyeong Kim, Dong Keon Yon.

**Writing – original draft**: Ye Won Kim, Juyeong Kim, Dong Keon Yon.

**Writing – review & editing**: Ye Won Kim, Juyeong Kim, Yesol Yim, Seoyoung Park, Hyunjee Kim, Lee Smith, André Hajek, Jiyoung Hwang, Dong Keon Yon.

## Supplementary Material
