# Supplementary material for: Long-term trends in anemia prevalence before and after COVID-19 among non-pregnant adults in South Korea, 2010 to 2023
Source: Medicine (Baltimore). 2026 May 12;104(49):e46295. doi: 10.1097/MD.0000000000046295 (PMC12688746; doi:10.1097/MD.0000000000046295)

**Table S1.** Weighted trends in the prevalence of anemia (weighted % [95% CI]) based on data obtained from the KNHANES, 2010–2023.

| Group | Pre-pandemic | | | Intra-pandemic | | | Post-pandemic |
| --- | --- | --- | --- | --- | --- | --- | --- |
|  | 2010–2012 | 2013–2016 | 2017–2019 | 2020 | 2021 | 2022 | 2023 |
| **Anemia** |  |  |  |  |  |  |  |
| **Overall** | 8.65 (8.11 to 9.19) | 7.99 (7.54 to 8.43) | 8.82 (8.30 to 9.34) | 11.72 (10.55 to 12.89) | 11.31 (10.22 to 12.40) | 10.67 (9.66 to 11.68) | 11.93 (10.86 to 13.00) |
| **Sex** |  |  |  |  |  |  |  |
| Male | 3.92 (3.37 to 4.46) | 4.03 (3.58 to 4.48) | 4.97 (4.40 to 5.55) | 6.89 (5.64 to 8.14) | 7.26 (5.98 to 8.55) | 6.78 (5.60 to 7.95) | 8.10 (6.96 to 9.25) |
| Female | 13.43 (12.54 to 14.31) | 12.05 (11.31 to 12.78) | 12.72 (11.91 to 13.53) | 16.51 (14.95 to 18.07) | 15.34 (13.57 to 17.11) | 14.57 (13.06 to 16.09) | 15.76 (14.05 to 17.47) |
| **Age** |  |  |  |  |  |  |  |
| 19-29 | 6.35 (5.11 to 7.58) | 4.15 (3.33 to 4.97) | 4.52 (3.52 to 5.51) | 4.87 (3.05 to 6.69) | 6.71 (4.23 to 9.18) | 6.87 (4.58 to 9.16) | 5.49 (3.73 to 7.25) |
| 30-39 | 7.98 (6.90 to 9.05) | 7.33 (6.39 to 8.28) | 7.06 (5.92 to 8.21) | 9.03 (6.58 to 11.48) | 7.35 (4.84 to 9.86) | 7.86 (5.77 to 9.96) | 9.08 (6.92 to 11.24) |
| 40-49 | 9.80 (8.58 to 11.02) | 9.66 (8.65 to 10.67) | 10.97 (9.66 to 12.27) | 12.27 (9.77 to 14.77) | 10.50 (8.10 to 12.89) | 11.09 (8.85 to 13.32) | 13.04 (10.80 to 15.28) |
| 50-59 | 5.67 (4.66 to 6.69) | 5.51 (4.71 to 6.32) | 5.21 (4.30 to 6.12) | 7.77 (5.65 to 9.89) | 7.31 (5.17 to 9.45) | 6.32 (4.51 to 8.12) | 8.22 (6.34 to 10.09) |
| 60-69 | 8.82 (7.49 to 10.15) | 8.79 (7.56 to 10.03) | 8.60 (7.38 to 9.83) | 11.26 (8.70 to 13.83) | 11.52 (9.10 to 13.94) | 9.64 (7.73 to 11.55) | 13.13 (11.20 to 15.07) |
| ≥70 | 18.71 (16.80 to 20.61) | 17.26 (15.66 to 18.86) | 20.20 (18.49 to 21.91) | 28.99 (24.98 to 33.01) | 27.91 (24.54 to 31.27) | 25.16 (21.55 to 28.78) | 24.59 (21.63 to 27.55) |
| **Region of residence** |  |  |  |  |  |  |  |
| Urban | 8.17 (7.44 to 8.90) | 7.82 (7.17 to 8.47) | 8.52 (7.77 to 9.28) | 11.51 (10.06 to 12.97) | 10.56 (9.26 to 11.85) | 10.18 (8.66 to 11.71) | 12.16 (10.37 to 13.95) |
| Rural | 9.10 (8.31 to 9.88) | 8.14 (7.53 to 8.75) | 9.12 (8.40 to 9.83) | 11.90 (10.11 to 13.70) | 12.01 (10.29 to 13.72) | 11.06 (9.73 to 12.39) | 11.74 (10.46 to 13.01) |
| **BMI group^*^** |  |  |  |  |  |  |  |
| Underweight | 13.13 (10.21 to 16.05) | 11.64 (9.34 to 13.94) | 11.40 (8.56 to 14.24) | 14.83 (8.86 to 20.79) | 15.96 (10.38 to 21.54) | 18.70 (12.32 to 25.08) | 24.04 (18.20 to 29.88) |
| Normal weight | 11.21 (10.32 to 12.10) | 10.43 (9.60 to 11.27) | 11.61 (10.73 to 12.48) | 14.17 (12.31 to 16.03) | 14.03 (11.91 to 16.14) | 13.37 (11.74 to 14.99) | 14.27 (12.48 to 16.06) |
| Overweight | 7.02 (6.05 to 8.00) | 6.51 (5.75 to 7.28) | 7.31 (6.33 to 8.30) | 12.23 (9.58 to 14.87) | 11.04 (8.94 to 13.15) | 10.42 (8.27 to 12.57) | 10.00 (8.19 to 11.80) |
| Obese | 5.89 (5.10 to 6.68) | 5.72 (5.11 to 6.33) | 6.20 (5.51 to 6.89) | 8.15 (6.65 to 9.65) | 7.88 (6.56 to 9.20) | 6.58 (5.41 to 7.74) | 8.76 (7.56 to 9.96) |
| Unknown | 8.59 (0.00 to 18.13) | 12.15 (0.00 to 25.28) | 20.04 (8.21 to 31.86) | 31.31 (21.41 to 41.21) | 22.66 (9.49 to 35.83) | 28.82 (14.95 to 42.68) | 23.54 (13.34 to 33.73) |
| **Level of education** |  |  |  |  |  |  |  |
| High school or lower education | 9.57 (8.83 to 10.31) | 9.52 (8.83 to 10.20) | 10.29 (9.49 to 11.09) | 14.60 (12.72 to 16.49) | 13.87 (12.15 to 15.60) | 12.74 (11.20 to 14.29) | 15.19 (13.59 to 16.78) |
| College or higher education | 7.31 (6.57 to 8.06) | 6.25 (5.66 to 6.84) | 7.29 (6.61 to 7.97) | 8.38 (7.13 to 9.62) | 8.56 (7.06 to 10.05) | 8.49 (7.08 to 9.90) | 8.79 (7.59 to 9.99) |
| Unknown | 11.25 (7.13 to 15.37) | 9.24 (7.49 to 10.98) | 11.62 (8.93 to 14.30) | 19.42 (15.07 to 23.77) | 18.08 (12.83 to 23.33) | 25.96 (17.41 to 34.52) | 34.81 (24.98 to 44.65) |
| **Household income** |  |  |  |  |  |  |  |
| Lowest quartile | 12.88 (11.38 to 14.38) | 11.07 (9.94 to 12.20) | 14.91 (13.55 to 16.27) | 21.57 (17.76 to 25.39) | 22.24 (18.93 to 25.56) | 18.01 (15.22 to 20.80) | 20.41 (17.10 to 23.72) |
| Second quartile | 8.44 (7.50 to 9.39) | 8.95 (8.04 to 9.87) | 9.11 (8.03 to 10.18) | 13.09 (10.72 to 15.47) | 11.87 (9.69 to 14.04) | 11.51 (9.33 to 13.69) | 13.83 (11.80 to 15.87) |
| Third quartile | 8.05 (7.11 to 9.00) | 7.13 (6.41 to 7.85) | 7.93 (7.04 to 8.81) | 9.61 (7.95 to 11.26) | 9.11 (7.32 to 10.90) | 9.13 (7.47 to 10.79) | 9.83 (8.11 to 11.55) |
| Highest quartile | 7.15 (6.20 to 8.09) | 6.61 (5.89 to 7.33) | 6.62 (5.79 to 7.45) | 8.22 (6.44 to 9.99) | 8.13 (6.35 to 9.92) | 7.95 (6.41 to 9.48) | 8.58 (7.22 to 9.94) |
| **Smoking status** |  |  |  |  |  |  |  |
| Current smoker | 2.18 (1.61 to 2.76) | 2.31 (1.80 to 2.81) | 3.33 (2.62 to 4.04) | 4.19 (2.70 to 5.68) | 3.87 (2.42 to 5.32) | 4.33 (2.95 to 5.71) | 5.62 (3.87 to 7.37) |
| Ex-smoker | 9.64 (8.40 to 10.88) | 9.08 (8.02 to 10.15) | 9.89 (8.76 to 11.01) | 13.25 (11.04 to 15.45) | 13.44 (11.17 to 15.70) | 13.15 (11.03 to 15.27) | 15.79 (13.53 to 18.06) |
| Non-smoker | 11.27 (10.44 to 12.09) | 9.85 (9.21 to 10.49) | 10.22 (9.51 to 10.94) | 13.41 (11.99 to 14.82) | 12.57 (11.08 to 14.07) | 11.23 (9.97 to 12.49) | 11.85 (10.44 to 13.26) |
| Unknown | 10.71 (6.67 to 14.74) | 8.27 (6.10 to 10.44) | 16.32 (9.31 to 23.33) | 23.84 (6.67 to 41.00) | 29.34 (10.89 to 47.78) | 27.06 (9.45 to 44.68) | 36.25 (25.77 to 46.74) |
| **Household food security status** |  |  |  |  |  |  |  |
| Insufficient | 13.55 (9.76 to 17.35) | 12.05 (9.60 to 14.49) | 13.79 (10.11 to 17.46) | 21.04 (12.23 to 29.85) | 16.32 (9.54 to 23.10) | 16.37 (5.90 to 26.84) | 17.93 (6.56 to 29.31) |
| Sufficient | 8.47 (7.93 to 9.01) | 7.81 (7.36 to 8.26) | 8.71 (8.19 to 9.24) | 11.48 (10.34 to 12.62) | 11.19 (10.09 to 12.28) | 10.63 (9.62 to 11.63) | 11.86 (10.80 to 12.92) |
| **Dietary supplement use** |  |  |  |  |  |  |  |
| Yes | 8.88 (8.11 to 9.65) | 7.56 (6.95 to 8.17) | 8.55 (7.88 to 9.23) | 11.52 (10.27 to 12.77) | 10.71 (9.41 to 12.02) | 9.92 (8.71 to 11.14) | 11.79 (10.58 to 13.00) |
| No | 8.48 (7.76 to 9.20) | 8.33 (7.75 to 8.91) | 9.14 (8.36 to 9.92) | 12.09 (10.21 to 13.97) | 12.66 (10.87 to 14.44) | 12.40 (10.49 to 14.31) | 12.21 (10.42 to 14.00) |
| **Physical activity frequency** |  |  |  |  |  |  |  |
| No | 9.56 (8.91 to 10.20) | 8.71 (8.18 to 9.25) | 9.50 (8.87 to 10.12) | 12.61 (11.29 to 13.93) | 11.95 (10.69 to 13.21) | 11.65 (10.34 to 12.95) | 12.48 (11.17 to 13.79) |
| 1-3 times/week | 5.34 (4.47 to 6.22) | 5.72 (4.78 to 6.67) | 6.49 (5.39 to 7.59) | 6.86 (4.94 to 8.79) | 8.67 (6.35 to 11.00) | 5.43 (3.78 to 7.07) | 8.73 (6.71 to 10.75) |
| ≥4 times/week | 8.36 (6.55 to 10.16) | 5.77 (4.60 to 6.94) | 6.50 (5.12 to 7.88) | 8.43 (5.74 to 11.11) | 8.30 (5.68 to 10.93) | 11.44 (7.85 to 15.03) | 11.34 (8.95 to 13.73) |
| Unknown | 10.71 (6.69 to 14.72) | 9.00 (7.26 to 10.74) | 11.77 (9.08 to 14.46) | 19.70 (15.32 to 24.09) | 18.09 (12.86 to 23.33) | 12.37 (8.32 to 16.43) | 16.01 (12.19 to 19.83) |
| **Average sleep duration per day** |  |  |  |  |  |  |  |
| <6 hours | 10.45 (8.94 to 11.96) | 7.99 (6.97 to 9.01) | 11.52 (9.95 to 13.10) | 14.56 (11.44 to 17.68) | 14.10 (11.01 to 17.20) | 15.82 (12.63 to 19.00) | 14.05 (11.52 to 16.58) |
| 6-9 hours | 8.09 (7.49 to 8.69) | 7.89 (7.39 to 8.40) | 8.13 (7.56 to 8.70) | 10.71 (9.44 to 11.99) | 10.81 (9.63 to 12.00) | 9.76 (8.66 to 10.86) | 11.51 (10.38 to 12.63) |
| ≥9 hours | 10.86 (8.80 to 12.92) | 8.75 (7.42 to 10.07) | 10.32 (8.89 to 11.75) | 13.92 (11.35 to 16.50) | 11.34 (8.21 to 14.48) | 11.63 (7.87 to 15.40) | 12.31 (9.12 to 15.50) |

Abbreviations: BMI, body mass index; CI, confidence interval; KNHANES, Korea National Health and Nutrition Examination Survey.

*According to Asian-Pacific guidelines, BMI is divided into four groups: underweight (<18.5 kg/m^2^), normal weight (18.5–22.9 kg/m^2^), overweight (23.0–24.9 kg/m^2^), and obese (≥25.0 kg/m^2^).

**Table S2.** β-coefficients and β-differences in anemia trends across pre-, intra-, and post-pandemic periods (weighted % [95% CI]).

| Group | Trends in the pre-pandemic, β (95% CI) | Trends in intra-pandemic, β (95% CI) | Trends in the post-pandemic, β (95% CI) | β_diff_ between pre- and intra-pandemic (95% CI) | β_diff_ between intra- and post-pandemic (95% CI) |  |
| --- | --- | --- | --- | --- | --- | --- |
|  |  |  |  |  |  |  |
| **Anemia** |  |  |  |  |  |  |
| **Overall** | 0.010 (-0.027 to 0.048) | **0.050 (0.012 to 0.088)** | 0.125 (-0.025 to 0.276) | 0.040 (-0.013 to 0.093) | 0.075 (-0.080 to 0.231) |  |
| **Sex** |  |  |  |  |  |  |
| Male | **0.054 (0.014 to 0.093)** | **0.057 (0.014 to 0.100)** | 0.133 (-0.040 to 0.305) | 0.004 (-0.055 to 0.062) | 0.075 (-0.102 to 0.253) |  |
| Female | -0.033 (-0.093 to 0.027) | 0.042 (-0.015 to 0.099) | 0.119 (-0.108 to 0.346) | 0.075 (-0.008 to 0.158) | 0.077 (-0.157 to 0.311) |  |
| **Age** |  |  |  |  |  |  |
| 19-29 | -0.091 (-0.170 to -0.012) | **0.089 (0.009 to 0.169)** | -0.138 (-0.424 to 0.147) | **0.180 (0.067 to 0.292)** | -0.228 (-0.524 to 0.069) |  |
| 30-39 | -0.046 (-0.124 to 0.033) | 0.008 (-0.071 to 0.087) | 0.122 (-0.177 to 0.421) | 0.054 (-0.058 to 0.165) | 0.114 (-0.195 to 0.423) |  |
| 40-49 | 0.059 (-0.031 to 0.148) | -0.014 (-0.098 to 0.070) | 0.195 (-0.117 to 0.508) | -0.073 (-0.195 to 0.050) | 0.209 (-0.115 to 0.533) |  |
| 50-59 | -0.024 (-0.092 to 0.044) | 0.028 (-0.039 to 0.096) | 0.190 (-0.066 to 0.446) | 0.052 (-0.044 to 0.148) | 0.162 (-0.103 to 0.427) |  |
| 60-69 | -0.011 (-0.101 to 0.079) | 0.026 (-0.053 to 0.104) | **0.349 (0.082 to 0.617)** | 0.037 (-0.083 to 0.157) | **0.324 (0.045 to 0.603)** |  |
| ≥70 | 0.091 (-0.036 to 0.219) | 0.122 (-0.012 to 0.255) | -0.057 (-0.523 to 0.408) | 0.031 (-0.154 to 0.215) | -0.179 (-0.663 to 0.305) |  |
| **Region of residence** |  |  |  |  |  |  |
| Urban | 0.019 (-0.034 to 0.071) | 0.042 (-0.012 to 0.096) | 0.197 (-0.035 to 0.430) | 0.023 (-0.052 to 0.099) | 0.156 (-0.083 to 0.394) |  |
| Rural | 0.003 (-0.050 to 0.056) | **0.056 (0.003 to 0.108)** | 0.067 (-0.129 to 0.264) | 0.053 (-0.022 to 0.128) | 0.012 (-0.192 to 0.215) |  |
| **BMI group^*^** |  |  |  |  |  |  |
| Underweight | -0.087 (-0.290 to 0.117) | **0.229 (0.003 to 0.455)** | 0.535 (-0.332 to 1.402) | **0.316 (0.011 to 0.620)** | 0.306 (-0.590 to 1.202) |  |
| Normal weight | 0.021 (-0.041 to 0.084) | 0.053 (-0.008 to 0.114) | 0.090 (-0.154 to 0.335) | 0.032 (-0.056 to 0.119) | 0.037 (-0.215 to 0.289) |  |
| Overweight | 0.016 (-0.053 to 0.085) | 0.079 (-0.001 to 0.158) | -0.042 (-0.324 to 0.240) | 0.063 (-0.042 to 0.168) | -0.121 (-0.414 to 0.172) |  |
| Obese | 0.017 (-0.036 to 0.069) | 0.006 (-0.040 to 0.052) | **0.218 (0.046 to 0.390)** | -0.010 (-0.080 to 0.059) | **0.212 (0.034 to 0.390)** |  |
| Unknown | 0.581 (-0.185 to 1.347) | 0.037 (-0.620 to 0.695) | -0.528 (-2.228 to 1.172) | -0.544 (-1.553 to 0.465) | -0.565 (-2.388 to 1.257) |  |
| **Level of education** |  |  |  |  |  |  |
| High school or lower education | 0.035 (-0.019 to 0.090) | **0.068 (0.010 to 0.126)** | **0.244 (0.025 to 0.464)** | 0.032 (-0.047 to 0.112) | 0.176 (-0.051 to 0.403) |  |
| College or higher education | 0.004 (-0.046 to 0.054) | 0.037 (-0.015 to 0.088) | 0.030 (-0.154 to 0.215) | 0.033 (-0.039 to 0.105) | -0.007 (-0.198 to 0.185) |  |
| Unknown | 0.063 (-0.171 to 0.296) | **0.378 (0.129 to 0.627)** | 0.885 (-0.403 to 2.174) | 0.316 (-0.025 to 0.657) | 0.507 (-0.805 to 1.820) |  |
| **Household income** |  |  |  |  |  |  |
| Lowest quartile | **0.106 (0.005 to 0.207)** | 0.091 (-0.018 to 0.200) | 0.240 (-0.189 to 0.668) | -0.015 (-0.164 to 0.134) | 0.149 (-0.294 to 0.591) |  |
| Second quartile | 0.033 (-0.038 to 0.104) | 0.062 (-0.017 to 0.141) | 0.232 (-0.060 to 0.525) | 0.029 (-0.078 to 0.135) | 0.170 (-0.133 to 0.473) |  |
| Third quartile | -0.005 (-0.070 to 0.060) | 0.030 (-0.031 to 0.092) | 0.070 (-0.166 to 0.307) | 0.035 (-0.054 to 0.125) | 0.040 (-0.205 to 0.285) |  |
| Highest quartile | -0.025 (-0.087 to 0.038) | 0.038 (-0.020 to 0.096) | 0.063 (-0.144 to 0.270) | 0.063 (-0.023 to 0.148) | 0.025 (-0.190 to 0.240) |  |
| **Smoking status** |  |  |  |  |  |  |
| Current smoker | **0.057 (0.011 to 0.102)** | 0.027 (-0.023 to 0.077) | 0.129 (-0.095 to 0.354) | -0.030 (-0.097 to 0.038) | 0.102 (-0.128 to 0.332) |  |
| Ex-smoker | 0.015 (-0.069 to 0.098) | **0.095 (0.015 to 0.174)** | 0.264 (-0.054 to 0.583) | 0.080 (-0.035 to 0.195) | 0.170 (-0.159 to 0.498) |  |
| Non-smoker | -0.049 (-0.104 to 0.005) | 0.021 (-0.028 to 0.069) | 0.062 (-0.127 to 0.250) | 0.070 (-0.003 to 0.143) | 0.041 (-0.154 to 0.236) |  |
| Unknown | 0.164 (-0.209 to 0.536) | 0.394 (-0.199 to 0.987) | 0.919 (-1.125 to 2.963) | 0.230 (-0.470 to 0.931) | 0.525 (-1.604 to 2.654) |  |
| **Household food security status** |  |  |  |  |  |  |
| Insufficient | -0.001 (-0.266 to 0.264) | 0.052 (-0.260 to 0.364) | 0.157 (-1.372 to 1.685) | 0.053 (-0.356 to 0.462) | 0.105 (-1.456 to 1.665) |  |
| Sufficient | 0.014 (-0.023 to 0.052) | **0.053 (0.015 to 0.091)** | 0.123 (-0.026 to 0.273) | 0.039 (-0.014 to 0.092) | 0.070 (-0.084 to 0.225) |  |
| **Dietary supplement use** |  |  |  |  |  |  |
| Yes | -0.010 (-0.061 to 0.041) | 0.024 (-0.023 to 0.071) | **0.187 (0.014 to 0.359)** | 0.034 (-0.036 to 0.103) | 0.163 (-0.016 to 0.342) |  |
| No | 0.032 (-0.021 to 0.085) | **0.112 (0.048 to 0.176)** | -0.019 (-0.284 to 0.246) | 0.080 (-0.003 to 0.163) | -0.131 (-0.404 to 0.141) |  |
| **Physical activity frequency** |  |  |  |  |  |  |
| No | -0.001 (-0.046 to 0.044) | **0.059 (0.011 to 0.106)** | 0.083 (-0.101 to 0.267) | 0.060 (-0.005 to 0.125) | 0.025 (-0.165 to 0.214) |  |
| 1-3 times/week | 0.057 (-0.013 to 0.126) | -0.016 (-0.083 to 0.050) | **0.331 (0.073 to 0.589)** | -0.073 (-0.169 to 0.023) | **0.347 (0.080 to 0.613)** |  |
| ≥4 times/week | -0.084 (-0.196 to 0.028) | **0.147 (0.024 to 0.269)** | -0.009 (-0.450 to 0.432) | **0.230 (0.065 to 0.396)** | -0.156 (-0.614 to 0.302) |  |
| Unknown | 0.100 (-0.131 to 0.331) | -0.058 (-0.235 to 0.119) | 0.364 (-0.201 to 0.929) | -0.158 (-0.449 to 0.133) | 0.422 (-0.170 to 1.014) |  |
| **Average sleep duration per day** |  |  |  |  |  |  |
| <6 hours | 0.057 (-0.052 to 0.166) | **0.124 (0.010 to 0.238)** | -0.177 (-0.590 to 0.236) | 0.067 (-0.091 to 0.225) | -0.300 (-0.729 to 0.128) |  |
| 6-9 hours | 0.002 (-0.039 to 0.044) | **0.049 (0.007 to 0.090)** | **0.175 (0.017 to 0.333)** | 0.046 (-0.012 to 0.104) | 0.126 (-0.037 to 0.289) |  |
| ≥9 hours | -0.014 (-0.136 to 0.109) | 0.008 (-0.120 to 0.135) | 0.068 (-0.423 to 0.558) | 0.021 (-0.156 to 0.198) | 0.060 (-0.447 to 0.567) |  |

Abbreviations: BMI, body mass index; CI, confidence interval.

*According to Asian-Pacific guidelines, BMI is divided into four groups: underweight (<18.5 kg/m^2^), normal weight (18.5–22.9 kg/m^2^), overweight (23.0–24.9 kg/m^2^), and obese (≥25.0 kg/m^2^).

The β values were multiplied by 10 owing to their minimal number.

**Table S3.** Adjusted odds ratios in the trends of anemia from 2010 to 2023 (weighted % [95% CI]), based on data obtained from the KNHANES.

| Variables | 2013–2016 versus  2010–2012 (reference) | 2017–2019 versus  2013–2016 (reference) | 2020 versus 2017–2019 (reference) | 2021 versus 2020 (reference) | 2022 versus 2021 (reference) | 2023 versus 2022 (reference) |
| --- | --- | --- | --- | --- | --- | --- |
|  | aOR (95% CI) | aOR (95% CI) | aOR (95% CI) | aOR (95% CI) | aOR (95% CI) | aOR (95% CI) |
| **Overall** | **0.90 (0.82 to 0.99)** | **1.11 (1.02 to 1.22)** | **1.37 (1.21 to 1.56)** | 0.97 (0.83 to 1.13) | 0.95 (0.81 to 1.11) | 1.13 (0.97 to 1.32) |
| **Sex** |  |  |  |  |  |  |
| Male | 0.97 (0.81 to 1.17) | 1.14 (0.96 to 1.35) | **1.36 (1.08 to 1.72)** | 1.03 (0.78 to 1.35) | 0.92 (0.70 to 1.21) | 1.20 (0.92 to 1.57) |
| Female | **0.87 (0.79 to 0.97)** | 1.09 (0.99 to 1.21) | **1.37 (1.19 to 1.57)** | 0.94 (0.78 to 1.12) | 0.96 (0.80 to 1.16) | 1.10 (0.92 to 1.31) |
| **Age** |  |  |  |  |  |  |
| 19-29 | **0.63 (0.47 to 0.85)** | 1.11 (0.81 to 1.53) | 1.13 (0.69 to 1.83) | 1.45 (0.83 to 2.56) | 1.08 (0.60 to 1.94) | 0.78 (0.46 to 1.32) |
| 30-39 | 0.94 (0.76 to 1.17) | 1.00 (0.79 to 1.25) | 1.34 (0.95 to 1.91) | 0.80 (0.49 to 1.31) | 1.18 (0.72 to 1.91) | 1.18 (0.79 to 1.76) |
| 40-49 | 0.97 (0.80 to 1.17) | **1.20 (1.00 to 1.45)** | 1.14 (0.86 to 1.50) | 0.86 (0.59 to 1.23) | 1.11 (0.76 to 1.61) | 1.25 (0.91 to 1.73) |
| 50-59 | 0.97 (0.75 to 1.24) | 0.99 (0.78 to 1.25) | **1.59 (1.12 to 2.25)** | 0.96 (0.63 to 1.48) | 0.88 (0.57 to 1.37) | 1.30 (0.88 to 1.93) |
| 60-69 | 1.03 (0.82 to 1.30) | 1.01 (0.81 to 1.25) | **1.41 (1.05 to 1.89)** | 1.03 (0.72 to 1.47) | 0.81 (0.58 to 1.13) | **1.39 (1.05 to 1.83)** |
| ≥70 | 0.90 (0.76 to 1.06) | 1.26 (1.07 to 1.48) | **1.59 (1.26 to 2.01)** | 0.96 (0.73 to 1.25) | 0.90 (0.70 to 1.17) | 0.95 (0.73 to 1.24) |
| **Region of residence** |  |  |  |  |  |  |
| Urban | 0.89 (0.78 to 1.02) | 1.11 (0.97 to 1.27) | **1.40 (1.18 to 1.67)** | 0.97 (0.78 to 1.20) | 0.91 (0.72 to 1.15) | 1.25 (0.98 to 1.61) |
| Rural | 0.91 (0.80 to 1.03) | 1.11 (0.99 to 1.25) | **1.35 (1.13 to 1.62)** | 0.97 (0.78 to 1.20) | 0.99 (0.81 to 1.22) | 1.03 (0.86 to 1.24) |
| **BMI group^*^** |  |  |  |  |  |  |
| Underweight | 0.85 (0.60 to 1.22) | 0.88 (0.60 to 1.28) | 1.20 (0.65 to 2.21) | 1.29 (0.65 to 2.55) | 1.28 (0.69 to 2.37) | 1.29 (0.76 to 2.21) |
| Normal weight | 0.88 (0.77 to 1.00) | 1.11 (0.97 to 1.26) | **1.24 (1.04 to 1.47)** | 1.00 (0.79 to 1.26) | 0.99 (0.79 to 1.24) | 1.07 (0.87 to 1.31) |
| Overweight | 0.95 (0.78 to 1.15) | 1.12 (0.92 to 1.37) | **1.87 (1.38 to 2.52)** | 0.88 (0.63 to 1.24) | 0.86 (0.61 to 1.20) | 1.00 (0.73 to 1.37) |
| Obese | 0.97 (0.81 to 1.17) | 1.12 (0.95 to 1.33) | **1.41 (1.11 to 1.78)** | 0.97 (0.74 to 1.27) | 0.84 (0.64 to 1.11) | 1.35 (1.05 to 1.73) |
| Unknown | 0.22 (0.02 to 2.04) | 1.81 (0.33 to 10.06) | 2.55 (0.64 to 10.13) | 0.65 (0.27 to 1.60) | 1.21 (0.42 to 3.54) | 0.93 (0.37 to 2.35) |
| **Level of education** |  |  |  |  |  |  |
| High school or lower education | 0.97 (0.86 to 1.09) | 1.05 (0.94 to 1.18) | **1.57 (1.32 to 1.86)** | 0.89 (0.72 to 1.11) | 0.90 (0.74 to 1.10) | **1.21 (1.00 to 1.46)** |
| College or higher education | **0.77 (0.66 to 0.90)** | 1.14 (0.98 to 1.32) | 1.13 (0.93 to 1.38) | 1.02 (0.79 to 1.33) | 1.03 (0.79 to 1.35) | 1.02 (0.81 to 1.30) |
| Unknown | 0.89 (0.53 to 1.51) | 1.15 (0.80 to 1.66) | 1.42 (0.90 to 2.25) | >999.999 (>999.999 to >999.999) | 1.04 (0.59 to 1.83) | 1.54 (0.62 to 3.84) |
| **Household income** |  |  |  |  |  |  |
| Lowest quartile | **0.82 (0.69 to 0.98)** | **1.37 (1.17 to 1.62)** | **1.46 (1.14 to 1.88)** | 1.05 (0.77 to 1.42) | 0.82 (0.63 to 1.08) | 1.07 (0.82 to 1.41) |
| Second quartile | 1.03 (0.87 to 1.22) | 0.99 (0.82 to 1.18) | **1.48 (1.15 to 1.90)** | 0.85 (0.63 to 1.15) | 1.00 (0.74 to 1.35) | 1.22 (0.92 to 1.61) |
| Third quartile | **0.84 (0.70 to 0.99)** | 1.12 (0.95 to 1.33) | 1.21 (0.96 to 1.53) | 0.96 (0.71 to 1.30) | 1.03 (0.76 to 1.40) | 1.10 (0.83 to 1.47) |
| Highest quartile | 0.90 (0.75 to 1.08) | 1.03 (0.86 to 1.24) | 1.31 (0.99 to 1.73) | 1.00 (0.72 to 1.39) | 1.02 (0.74 to 1.40) | 1.09 (0.83 to 1.44) |
| **Smoking status** |  |  |  |  |  |  |
| Current smoker | 1.01 (0.70 to 1.45) | 1.36 (0.98 to 1.89) | 1.18 (0.76 to 1.83) | 1.00 (0.58 to 1.73) | 1.10 (0.65 to 1.88) | 1.32 (0.79 to 2.19) |
| Ex-smoker | 0.95 (0.78 to 1.17) | 1.04 (0.86 to 1.25) | **1.47 (1.17 to 1.84)** | 0.97 (0.73 to 1.28) | 1.10 (0.82 to 1.48) | 1.14 (0.87 to 1.51) |
| Non-smoker | **0.88 (0.78 to 0.98)** | 1.07 (0.96 to 1.19) | **1.37 (1.18 to 1.59)** | 0.95 (0.79 to 1.15) | 0.87 (0.72 to 1.05) | 1.12 (0.93 to 1.34) |
| Unknown | 0.85 (0.49 to 1.48) | 1.82 (0.99 to 3.36) | 0.73 (0.24 to 2.27) | >999.999 (>999.999 to >999.999) | 3.33 (0.45 to 24.49) | 2.04 (0.55 to 7.53) |
| **Household food security status** |  |  |  |  |  |  |
| Insufficient | 0.90 (0.59 to 1.38) | 0.86 (0.60 to 1.25) | 1.84 (0.98 to 3.46) | 0.69 (0.34 to 1.40) | 1.05 (0.40 to 2.80) | 0.66 (0.18 to 2.36) |
| sufficient | **0.90 (0.82 to 0.99)** | **1.12 (1.02 to 1.23)** | **1.35 (1.19 to 1.54)** | 0.98 (0.84 to 1.15) | 0.95 (0.81 to 1.11) | 1.14 (0.98 to 1.32) |
| **Dietary supplement use** |  |  |  |  |  |  |
| Yes | **0.82 (0.72 to 0.93)** | **1.15 (1.02 to 1.31)** | **1.38 (1.19 to 1.61)** | 0.93 (0.78 to 1.12) | 0.92 (0.76 to 1.12) | **1.22 (1.01 to 1.46)** |
| No | 0.98 (0.87 to 1.11) | 1.07 (0.95 to 1.21) | **1.37 (1.11 to 1.68)** | 1.04 (0.81 to 1.34) | 0.99 (0.77 to 1.28) | 0.98 (0.77 to 1.26) |
| **Physical activity frequency** |  |  |  |  |  |  |
| No | 0.92 (0.83 to 1.01) | 1.10 (0.99 to 1.21) | **1.43 (1.24 to 1.65)** | 0.91 (0.76 to 1.09) | 0.96 (0.80 to 1.15) | 1.08 (0.90 to 1.28) |
| 1-3 times/week | 0.98 (0.75 to 1.27) | 1.09 (0.84 to 1.41) | 1.13 (0.78 to 1.63) | 1.27 (0.82 to 1.96) | 0.57 (0.36 to 0.91) | **1.57 (1.03 to 2.40)** |
| ≥4 times/week | **0.64 (0.47 to 0.89)** | 1.09 (0.78 to 1.52) | 1.32 (0.88 to 1.99) | 0.92 (0.55 to 1.53) | 1.38 (0.85 to 2.24) | 0.93 (0.60 to 1.44) |
| Unknown | 0.88 (0.51 to 1.52) | 1.20 (0.83 to 1.73) | 1.47 (0.93 to 2.33) | 0.55 (<0.001 to >999.999) | 1.08 (0.61 to 1.92) | 1.42 (0.74 to 2.76) |
| **Average sleep duration per day** |  |  |  |  |  |  |
| <6 hours | **0.78 (0.63 to 0.98)** | **1.45 (1.16 to 1.82)** | 1.30 (0.95 to 1.76) | 0.95 (0.61 to 1.48) | 0.98 (0.68 to 1.40) | 0.94 (0.68 to 1.30) |
| 6-9 hours | 0.95 (0.85 to 1.06) | 1.03 (0.93 to 1.15) | **1.42 (1.22 to 1.65)** | 0.96 (0.80 to 1.15) | 0.91 (0.76 to 1.10) | **1.18 (1.00 to 1.40)** |
| ≥9 hours | **0.75 (0.58 to 0.99)** | 1.23 (0.97 to 1.57) | 1.24 (0.91 to 1.68) | 0.86 (0.54 to 1.37) | 1.31 (0.80 to 2.14) | 1.13 (0.71 to 1.80) |

Abbreviations: BMI, body mass index; CI, confidence interval; KNHANES, Korea National Health and Nutrition Examination Survey; aOR, adjusted odds ratio.

*According to Asian-Pacific guidelines, BMI is divided into four groups: underweight (<18.5 kg/m^2^), normal weight (18.5–22.9 kg/m^2^), overweight (23.0–24.9 kg/m^2^), and obese (≥25.0 kg/m^2^).

The figures in bold indicate statistical significance (p-value < 0.05).

The overall multivariable logistic regression model adjusted for sex (male and female), age (19–29, 30–39, 40–49, 50–59, 60–69, and 70≤ years), region of residence (urban and rural), BMI group (underweight, normal weight, overweight, obese, and unknown), level of education(high school or lower education, college or higher education, and unknown), household income(lowest quartile, second quartile, third quartile, and highest quartile), smoking status (current smoker, ex-smoker, non-smoker, and unknown), household food security status (insufficient and sufficient), dietary supplement use (yes and no), physical activity frequency (no, 1-3 times/week, ≥4 times/week, and unknown) and average sleep duration per day (<6, 6-9, and ≥9 hours).

**Table S4.** Sex-specific trends in the prevalence of anemia by age during the pre-, intra-, and post-pandemic periods (weighted % [95% CI]).

| Group | Total | Pre-pandemic | | | Intra-pandemic | | | Post-pandemic |
| --- | --- | --- | --- | --- | --- | --- | --- | --- |
|  |  | 2010–2012 | 2013–2016 | 2017–2019 | 2020 | 2021 | 2022 | 2023 |
| **Age** |  |  |  |  |  |  |  |  |
| **Male** |  |  |  |  |  |  |  |  |
| 19-29 | 0.84 (0.42 to 1.25) | 0.99 (0.00 to 2.03) | 0.68 (0.16 to 1.19) | 0.53 (0.00 to 1.05) | 0.61 (0.00 to 1.64) | 2.44 (0.18 to 4.71) | 0.30 (0.00 to 0.71) | 0.28 (0.00 to 0.83) |
| 30-39 | 0.80 (0.47 to 1.12) | 0.64 (0.12 to 1.15) | 0.74 (0.28 to 1.21) | 1.19 (0.43 to 1.94) | 0.37 (0.00 to 1.10) | 0.50 (0.00 to 1.26) | 0.90 (0.00 to 1.98) | 1.25 (0.00 to 2.70) |
| 40-49 | 2.25 (1.71 to 2.79) | 2.43 (1.33 to 3.54) | 1.56 (0.85 to 2.27) | 2.16 (1.25 to 3.07) | 3.54 (1.50 to 5.59) | 1.34 (0.00 to 2.77) | 1.87 (0.30 to 3.44) | 2.83 (1.06 to 4.60) |
| 50-59 | 4.80 (3.98 to 5.62) | 3.91 (2.60 to 5.21) | 4.18 (3.08 to 5.29) | 3.57 (2.41 to 4.73) | 6.16 (3.49 to 8.84) | 4.63 (1.92 to 7.33) | 4.94 (2.41 to 7.47) | 5.97 (3.53 to 8.42) |
| 60-69 | 11.03 (9.89 to 12.17) | 9.63 (7.63 to 11.64) | 9.85 (8.12 to 11.58) | 9.11 (7.26 to 10.97) | 9.19 (6.02 to 12.36) | 12.41 (8.73 to 16.09) | 10.75 (7.58 to 13.93) | 14.40 (11.22 to 17.57) |
| ≥70 | 26.76 (24.81 to 28.72) | 19.42 (16.54 to 22.31) | 18.93 (16.51 to 21.34) | 22.59 (20.05 to 25.13) | 28.68 (22.11 to 35.26) | 30.51 (25.04 to 35.98) | 28.96 (23.19 to 34.73) | 30.07 (25.39 to 34.75) |
| **Female** |  |  |  |  |  |  |  |  |
| 19-29 | 10.85 (9.57 to 12.12) | 12.20 (9.88 to 14.51) | 8.23 (6.60 to 9.86) | 9.15 (7.14 to 11.16) | 9.50 (5.87 to 13.14) | 11.36 (6.97 to 15.75) | 14.16 (9.64 to 18.69) | 11.27 (7.68 to 14.86) |
| 30-39 | 16.07 (14.73 to 17.42) | 16.04 (13.90 to 18.17) | 14.78 (12.96 to 16.60) | 13.79 (11.62 to 15.96) | 18.78 (13.89 to 23.66) | 15.03 (9.97 to 20.09) | 15.91 (11.97 to 19.85) | 18.41 (14.39 to 22.43) |
| 40-49 | 20.26 (18.98 to 21.55) | 17.61 (15.46 to 19.77) | 18.16 (16.26 to 20.06) | 20.10 (17.74 to 22.46) | 21.40 (17.41 to 25.39) | 20.17 (15.52 to 24.82) | 20.79 (16.74 to 24.84) | 23.69 (19.73 to 27.65) |
| 50-59 | 8.45 (7.50 to 9.40) | 7.45 (5.99 to 8.91) | 6.87 (5.62 to 8.11) | 6.88 (5.45 to 8.32) | 9.38 (6.18 to 12.58) | 10.03 (6.85 to 13.22) | 7.70 (4.93 to 10.47) | 10.47 (7.83 to 13.11) |
| 60-69 | 10.07 (9.09 to 11.05) | 8.06 (6.32 to 9.80) | 7.89 (6.42 to 9.37) | 8.18 (6.57 to 9.80) | 12.84 (9.33 to 16.36) | 10.80 (7.72 to 13.88) | 8.71 (6.37 to 11.05) | 12.09 (9.86 to 14.32) |
| ≥70 | 21.87 (20.43 to 23.31) | 18.22 (15.68 to 20.76) | 15.95 (13.74 to 18.16) | 18.21 (16.02 to 20.41) | 29.27 (24.80 to 33.74) | 25.66 (21.44 to 29.88) | 21.90 (17.92 to 25.89) | 19.72 (15.99 to 23.46) |

Abbreviations: CI, confidence interval.

**Figure S1.** Study population flowchart.


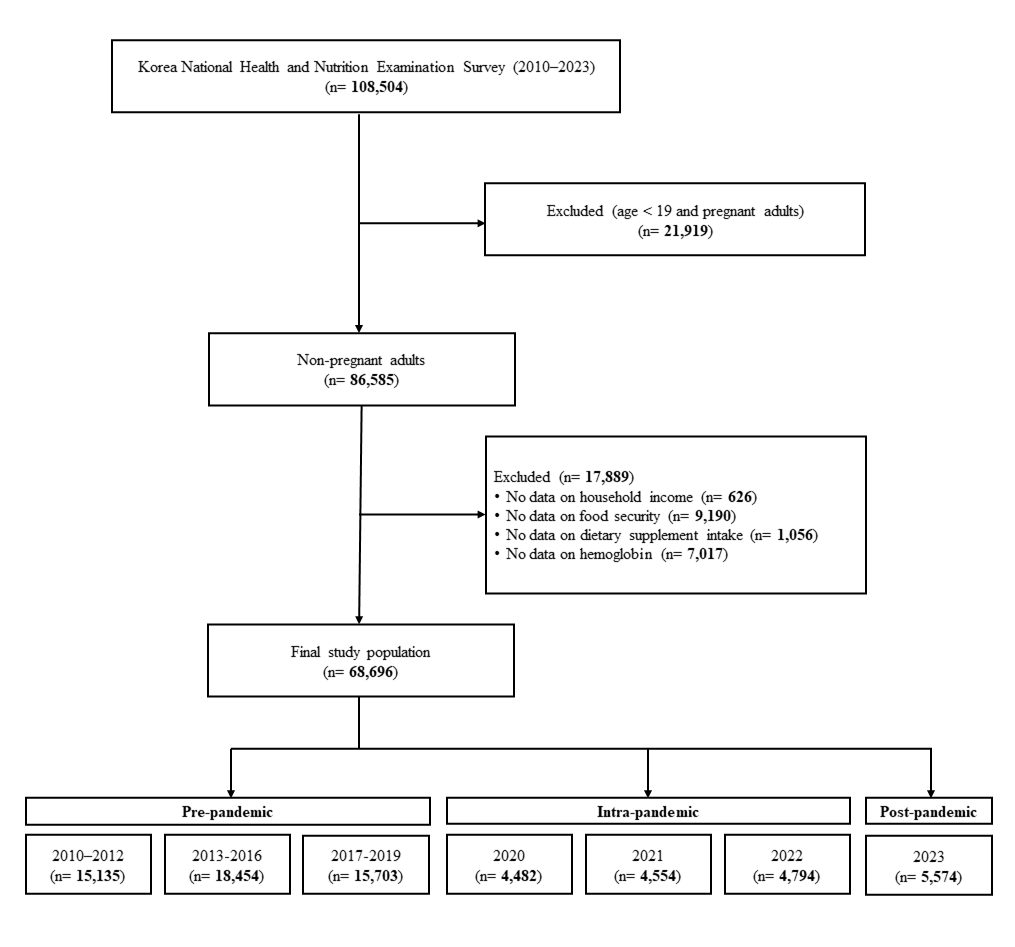


**Figure S2.** Trends in anemia prevalence during the pre-, intra-, and post-pandemic from 2010 to 2023 (n= 68,696). The error bars indicate the 95% confidence intervals.


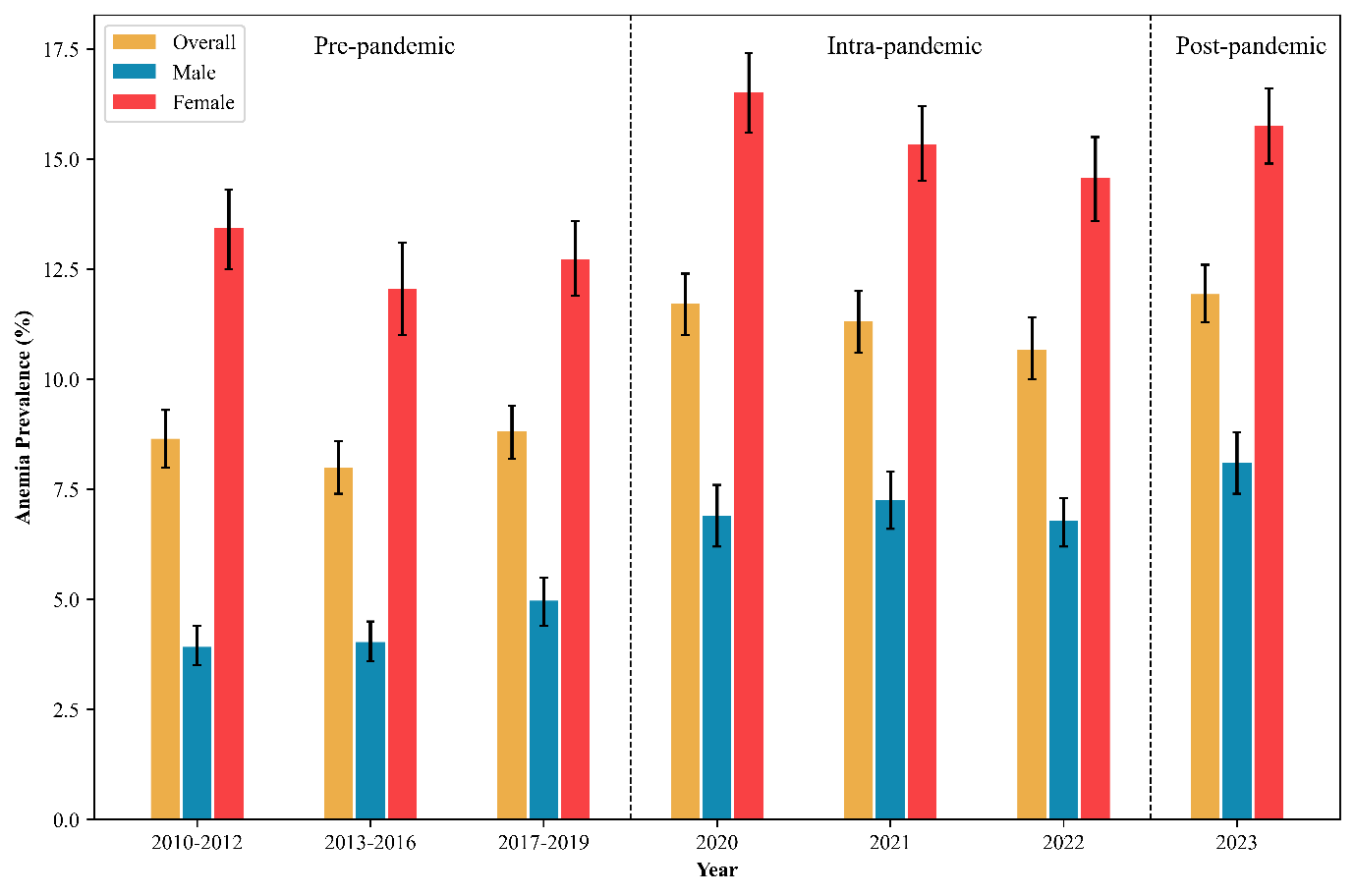

Supplement: Supplementary file 1 [file medi-104-e46295-s001.docx]
